# Correction to ‘Complex ecological interactions across a focus of cutaneous leishmaniasis in Eastern Colombia: novel description of *Leishmania* species, hosts and phlebotomine fauna’

**DOI:** 10.1098/rsos.201328

**Published:** 2020-08-26

**Authors:** Claudia M. Sandoval-Ramírez, Carolina Hernández, Aníbal A. Teherán, Reinaldo Gutierrez-Marin, Ruth A. Martínez-Vega, Duvan Morales, Richard Hoyos-Lopez, Astrid Araque-Mogollón, Juan David Ramírez

*R. Soc. Open Sci.* **7**, 200266. (Published online 8 July 2020). (doi:10.1098/rsos.200266)

This correction has been published due to an error in a collection permission of biological specimens in the Methods section. In §2.1 Study area, it was stated that:

Universidad del Rosario provided the field permit from ANLA (Autoridad Nacional de Licencias Ambientales) 63257–2014. All collections were done on public land.

This has been updated to:

Universidad de Santander (UDES) provided the collection permit from ANLA (Autoridad Nacional de Licencias Ambientales) 01749. All collections were done on public land.

