# Supplementary figures and images for: Complex ecological interactions across a focus of cutaneous leishmaniasis in Eastern Colombia: novel description of Leishmania species, hosts and phlebotomine fauna
Source: R Soc Open Sci. 2020 Jul 8;7(7):200266. doi: 10.1098/rsos.200266 (PMC7428272; doi:10.1098/rsos.200266)

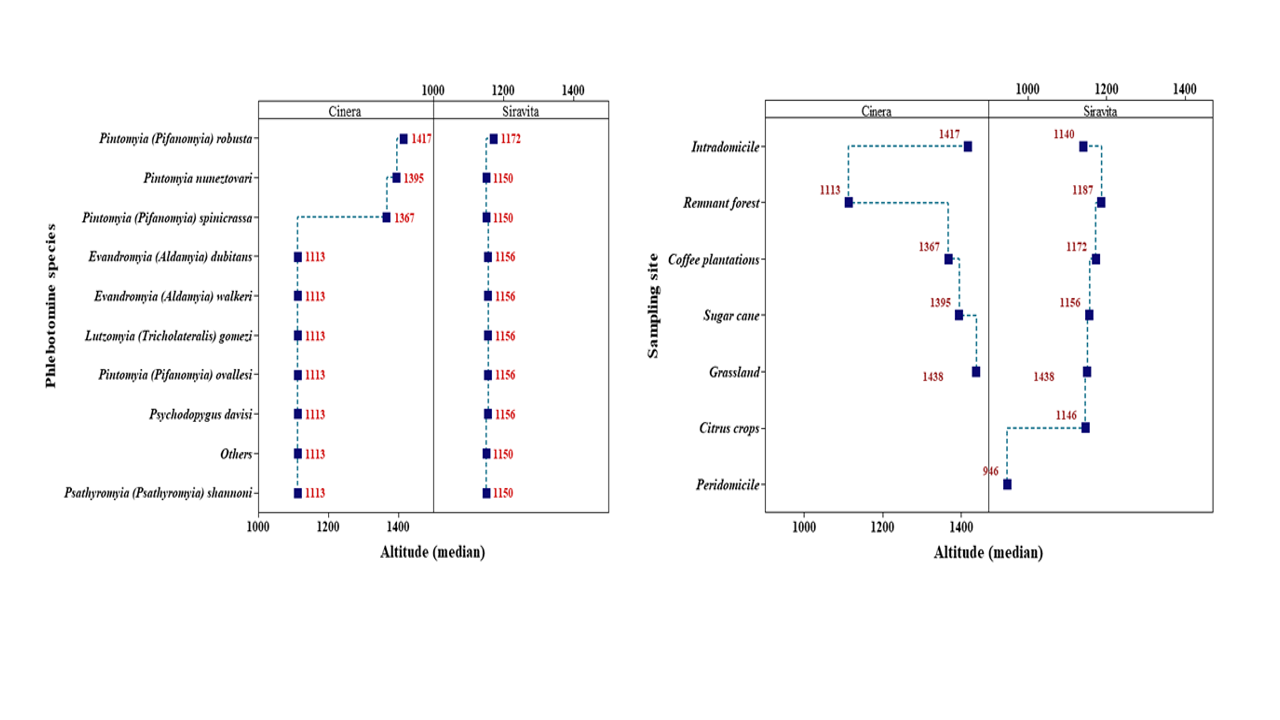

Supplement: S1 Fig. [file rsos200266supp1.tif]

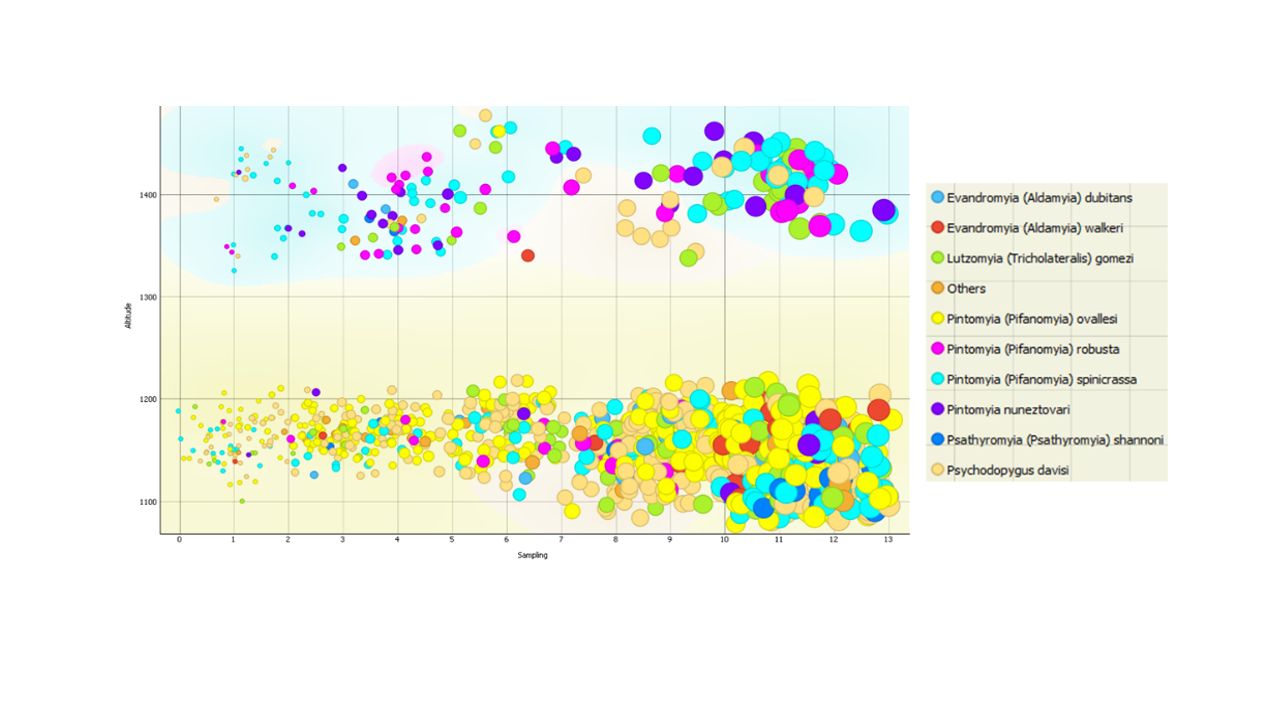

Supplement: S2 Fig. [file rsos200266supp2.tif]

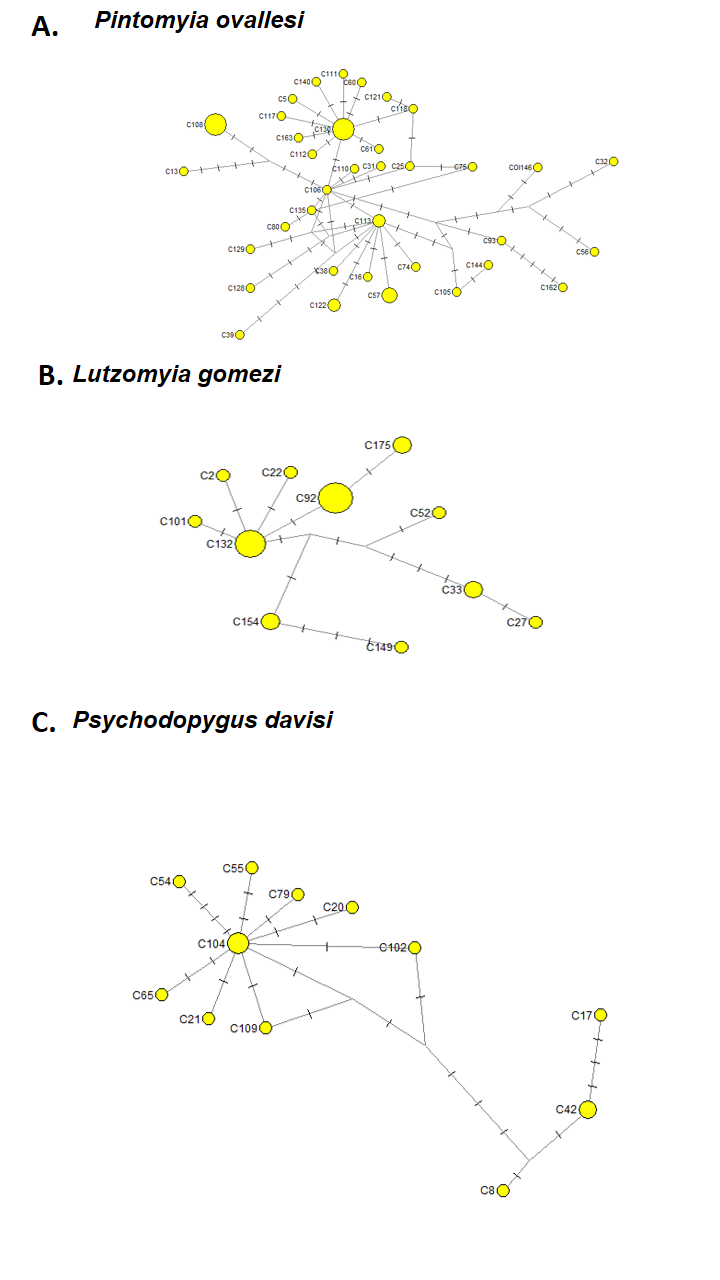

Supplement: Fig S3. [file rsos200266supp3.tif]
