# Supplementary material for: Complex ecological interactions across a focus of cutaneous leishmaniasis in Eastern Colombia: novel description of Leishmania species, hosts and phlebotomine fauna
Source: R Soc Open Sci. 2020 Jul 8;7(7):200266. doi: 10.1098/rsos.200266 (PMC7428272; doi:10.1098/rsos.200266)
Supplement: Table S1. [file rsos200266supp4.docx]

**Table S1. Species composition, total abundance, and relative abundance (%) of male and female phlebotomine sandflies caught in Cinera and Siravita, Norte de Santander, Colombia.**

| **Species** | **Locality** | | | | **Total (%)** |
| --- | --- | --- | --- | --- | --- |
|  | **Cinera** | | **Siravita** | |  |
|  | **Females** | **Males** | **Females** | **Males** |  |
| *Brumptomyia beaupertuyi* | 1 | 0 | 0 | 1 | 2 (0.12) |
| *Evandromyia (Aldamyia) dubitans* | 5 | 3 | 22 | 8 | 38 (2.20) |
| *Evandromyia (Aldamyia) walkeri* | 1 | 3 | 17 | 10 | 31 (1.79) |
| *Lutzomyia (Helcocyrtomyia) erwindonaldoi* | 1 | 2 | 4 | 1 | 8 (0.46) |
| *Lutzomyia (Helcocyrtomyia) scorzai* | 2 | 0 | 1 | 0 | 3 (0.17) |
| *Lutzomyia (Lutzomyia) lichyi* | 0 | 0 | 3 | 0 | 3 (0.17) |
| *Lutzomyia (Tricholateralis) gomezi* | 166 | 11 | 68 | 28 | 273 (15.80) |
| *Lutzomyia (Helcocyrtomyia) hartmanni* | 0 | 0 | 5 | 0 | 5 (0.29) |
| *Nyssomyia* sp. | 0 | 0 | 2 | 0 | 2 (0.12) |
| *Micropygomyia* sp. | 0 | 0 | 3 | 0 | 3 (0.17) |
| *Pintomyia (Pifanomyia) ovallesi* | **50** | 1 | **398** | 65 | 514 (29.75) |
| *Pintomyia (Pifanomyia) pia* | 2 | 0 | 3 | 1 | 6 (0.35) |
| *Pintomyia (Pifanomyia) robusta* | 31 | 16 | 11 | 7 | 65 (3.76) |
| *Pintomyia (Pifanomyia) spinicrassa* | 154 | 19 | 120 | 27 | 320 (18.52) |
| *Pintomyia nuneztovari* | 36 | 3 | 5 | 3 | 47 (2.72) |
| *Psathyromyia (Psathyromyia) shannoni* | 33 | 14 | 3 | 2 | 52 (3.01) |
| *Psychodopygus davisi* | 75 | 22 | 153 | 101 | 351 (20.31) |
| *Trichopygomyia ferroae* | 1 | 0 | 3 | 1 | 5 (0.29) |
| **Number of specimens** | **558** | **94** | **822** | **255** | **1729** |
| **Number of species** | **14** | | **17** | | **18** |
| Diversity (Order q = 0) | **15.00** (11.17–21.68) | | **20.00** (15.94–30.95) | | **18** (16.37–20.42) |
| Diversity (Order q = 1) | **6.78** (6.31–7.23)^a^ | | **5.32** (4.97–5.67) | | **6.71** (6.43–7.02) |
| Diversity (Order q = 2) | **5.40** (5.01–5.75)^a^ | | **3.72** (3.47–3.95) | | **5.20** (5.01–5.41) |
| Coverage (q = 0, 1, and 2) | **100** (100–100) | | **100** (100–100) | | **100** (100–100) |

Estimated diversity and sample coverage (q = 0, 1, and 2) with confidence intervals, based on a bootstrap method of 100 replications (0.95). ^a^ Statistically significant differences between localities.
