# Supplementary material for: Complex ecological interactions across a focus of cutaneous leishmaniasis in Eastern Colombia: novel description of Leishmania species, hosts and phlebotomine fauna
Source: R Soc Open Sci. 2020 Jul 8;7(7):200266. doi: 10.1098/rsos.200266 (PMC7428272; doi:10.1098/rsos.200266)
Supplement: Table S2 [file rsos200266supp5.docx]

**Table S2. Species composition and relative abundance of phlebotomine sandflies in each environment.**

| **Species** | **Environment** | | | | |
| --- | --- | --- | --- | --- | --- |
|  | **Forest remnants** | **Coffee plantations** | **Grassland** | **Cane plantations** | **Citrus plantations** |
| *Pintomyia (Pifanomyia) ovallesi* | 135 | 112 | 124 | 83 | 23 |
| *Psychodopygus davisi* | 162 | 50 | 81 | 25 | 19 |
| *Pintomyia (Pifanomyia) spinicrassa* | 37 | 76 | 37 | 12 | 15 |
| *Lutzomyia (Tricholateralis) gomezi* | 35 | 40 | 15 | 23 | 9 |
| *Pintomyia (Pifanomyia) robusta* | 7 | 41 | 5 | 0 | 1 |
| *Evandromyia (Aldamyia) dubitans* | 9 | 7 | 3 | 13 | 5 |
| *Pintomyia nuneztovari* | 13 | 16 | 4 | 2 | 1 |
| *Evandromyia (Aldamyia) walkeri* | 7 | 4 | 1 | 17 | 2 |
| *Psathyromyia (Psathyromyia) shannoni* | 11 | 3 | 3 | 2 | 0 |
| *Lutzomyia (Helcocyrtomyia) erwindonaldoi* | 3 | 2 | 0 | 1 | 0 |
| *Lutzomyia hartmanni* | 1 | 0 | 2 | 2 | 0 |
| *Trichopygomyia ferroae* | 3 | 0 | 0 | 0 | 1 |
| *Lutzomyia (Lutzomyia) lichyi* | 1 | 1 | 1 | 0 | 0 |
| *Pintomyia (Pifanomyia) pia* | 0 | 3 | 0 | 0 | 0 |
| *Brumptomyia beaupertuyi* | 1 | 0 | 0 | 1 | 0 |
| *Nyssomyia* sp. | 0 | 0 | 0 | 0 | 2 |
| *Micropygomyia* sp. | 2 | 0 | 0 | 0 | 0 |
| Number specimens | **427** | **355** | **276** | **181** | **78** |
| Number species | **15** | **12** | **11** | **12** | **10** |
| Diversity (q = 0) | 19.49 (13.34–29.86) | 12.5 (9.74–17.77) | 12.99 (9.5–18.61) | 11.66 (8.23–19.47) | 12.22 (7.77–21.66) |
| Diversity (q = 1) | 5.62 (5.11–6.24)* | 6.53 (5.96–7.12)* | 4.39 (3.96–4.99) | 5.6 (4.8–6.28) | 6.57 (5.49–7.99)* |
| Diversity (q = 2) | 3.86 (3.5–4.28) | 5.22 (4.62–5.16)* | 3.25 (2.95–3.67) | 3.84 (3.11–4.62) | 5.2 (4.29–6.17)* |
| Coverage (q = 0) | 98 (97–99) | 99 (98–99) | 98 (97–99) | 98 (98–100) | 99 (96–101) |
| Coverage (q = 1) | 98 (97–99) | 99 (98–99) | 98 (97–99) | 98 (98–100) | 99 (97–101) |
| Coverage (q = 2) | 98 (97–99) | 99 (98–99) | 98 (97–99) | 98 (98–100) | 99 (96–101) |

Estimated diversity profiles and coverage-based rarefaction and extrapolation for diversity (q = 0, 1, and 2) with 95% confidence intervals. ^a^Statistically significant differences between pasture and other environments.
